# Editorial: Modulation of Ion Channels and Ionic Pumps by Fatty Acids: Implications in Physiology and Pathology

**DOI:** 10.3389/fphys.2018.01625

**Published:** 2018-11-26

**Authors:** Mario Diaz, Mauricio A. Retamal

**Affiliations:** ^1^Sección Biologia, Departamento de Biologia Animal, Edafología y Geología, Facultad de Ciencias, Universidad de La Laguna, Tenerife, Spain; ^2^Unidad Asociada de Investigacion CSIC-ULL “Fisiología y Biofísica de la Membrana Celular en Patologías Neurodegenerativas y Tumorales”, Tenerife, Spain; ^3^Centro de Fisiología Celular e Integrativa, Clínica Alemana, Universidad del Desarrollo, Santiago, Chile; ^4^Department of Cell Physiology and Molecular Biophysics, Center for Membrane Protein Research, Texas Tech University Health Sciences Center, Lubbock, TX, United States

**Keywords:** fatty acids, ion channels, ionic pumps, PUFAs, plasma membrane

Polyunsaturated fatty acids (PUFAs) are fatty acids with two or more unsaturations. They are essential components of membrane phospholipids and it is well-known that under physiological conditions, PUFAs have a big impact in the human health. There exist two major series of PUFAs, i.e., omega-3 (or n-3 PUFA) and omega-6 (or n-6 PUFA), depending on the positions of the first double bond. The balance between n-6 PUFA and n-3 PUFA, is critical and associated to pathological conditions such cardiovascular and neurodegenerative diseases (Díaz and Marín, 2013; Dyall, 2017; Abdelhamid et al., 2018). In general, these fatty acids are incorporated into cell membrane phospholipids and are responsible for tuning a number of physicochemical properties of the plasma membrane, but are also the source for different bioactive lipids which have different effects on human health. Further, when free radicals are produced, PUFAs can be oxidized to lipid peroxides, which have profound effects on cell function, even inducing cell death (Catalá and Díaz, 2017). In spite of all these years of research, it is still not completely understood the molecular and cellular basis that rule the effects of PUFAs and lipid-derived peroxides on different cellular processes. Mounting evidence accumulated over the last decades indicate that many of their effects may be attributed to the modulation of membrane bilayer microenvironment, e.g., microdomains dynamics and a plethora of physicochemical properties, but also through direct interactions with membrane embedded proteins, these including ion channels, ionic pumps, transporters, neurotransmitter and hormone receptors, amongst other protein entities, at the plasma membrane which, in turn, will modify cellular contents of ions and metabolites, signaling and transduction pathways impacting the many faces of cell homeostasis (Cordero-Morales and Vásquez, 2018).

In this research topic we have gathered together 10 articles which contribute to the comprehension of the effects of fatty acids (PUFAs and other bioactive lipids) on ion channels and ionic pumps properties. The focus is put not only in the molecular interactions, but also on the systemic effects of their alterations in pathological conditions, as for example in the bipolar disorder (hypothesized in the article by Riveros and Retamal), which might pave the way for their understanding and to uncover new therapeutical directions.

In this Research topic, Antollini and Barrantes reviewed the effects of PUFAs on several types of voltage-gated K^+^, Na^+^, and anionic channels as well as in GABA and nicotinic receptors. Based on the current data they concluded that the effect of a given PUFA depend on the type of channel under consideration and, moreover, that similar PUFAs may cause diverse effects on very closely related channels from a phylogenetic point of view. However, it seems clear that almost all PUFAs that modulate voltage- and ligand-gated channel directly contact with them. Therefore, nowadays a hot topic in the field is the study of those amino acids that participate in the interaction with the PUFAs. Antollini and Barrantes also discuss some of the rules that govern the potential interaction between amino acid residues and PUFAs. Thus, the length, isomerism and saturation of the PUFAs seems to be very important factors. In this order of ideas, Elinder and Liin summarize data from different PUFAs on voltage-gated ion channels containing one or more voltage-sensor domains, such as voltage-gated sodium (Na_V_), potassium (K_V_), calcium (Ca_V_), and proton (H_V_) channels, as well as calcium-activated potassium (K_Ca_), and transient receptor potential (TRP) channels. In this very interesting work they propose that the effect of PUFAs on voltage gated ion channels can be grouped into three main categories (1) those causing alterations in voltage dependence, (2) those causing changes in the maximal conductance and (3) those causing changes in the gating kinetics. They also propose five molecular mechanism by which PUFAs can exert these effects: (1) by binding to amino acids located at the ion-conducting pore, (2) by binding to amino acids at the extracellular side of the channel forming an open-channel block, (3) by binding at the voltage sensor domain (VSD) linker close to the intracellular gate, (4) at the interface between the extracellular part of the channel and the external leaflet, from where they can electrostatically affect the S4 VSD and (5) by electrostatic effects on the pore domain. In agreement, in the article by Moreno et al. on K^+^ channels, the mechanism of modulation involves an electrostatic effect of PUFAs on VSD. These authors explain in detail that the negative charge of the carboxyl group of the fatty acid is crucial for the electrostatic interaction between PUFA and the VSD (at the bilayer/channel interface close to the S3-S4 segment). Similarly, in the original research article by Oliván-Viguera et al., the effects of omega-3 fatty acids on the calcium/calmodulin-gated KCa3.1 properties expressed in murine and human fibroblasts were analyzed. KCa3.1 channel function has been linked to abnormal cell proliferation, pathological tissue remodeling and fibrosis of a variety of organs, chronic inflammation, and autoimmune diseases. They found that α-linolenic acid and docosahexanenoic acid (DHA) inhibit KCa3.1 currents and strongly reduce fibroblast growth in a dose-dependent way. The association between KCa3.1 and PUFA as well as the link between KCa3.1 and human diseases, led them to suggest that reduced fibroblast KCa3.1 functions might be a feature and a possible biomarker of cell dysfunction in Lysosomal Storage Disorders.

PUFAs can also modulate ion channels activity through generation of bioactive lipids. In particular pro-resolvins have been shown to modulate different types of ion channels, including epithelial Na^+^ channels, cystic fibrosis transmembrane conductance regulators, ATP-sensitive K^+^ channels, Ca^2+^-activated anion channels, pannexin 1 hemichannels, and canonical subtype of TRP channels (TRPCs). In this RT, Choi and Hwang review the effects of various pro-resolving lipids on the functions of four sensory TRP channels and NMDA receptors and discussed their beneficial effects on inflammation and pain. Nowadays, the physiological significance of bioactive lipids is expanding and new roles in their signaling properties are growing rather slowly, perhaps, the main reason for this, is that these molecules are usually found in very small quantities and often they are co-extracted with other lipophilic molecules, making their detection and identification a hard task. In this RT, Divito et al. discuss on the fact that common analytical methodologies are usually ineffective due to the unsuccessful separation of bioactive lipids in complex biological samples, and show that novel multidimensional separation techniques are very useful separation strategies for these elusive molecules.

Several articles are included in this RT showing different forms of regulation of Cx46 hemichannels by lipid molecules. In this Research Topic, Retamal presents evidence supporting the hypothesis that carbon monoxide inhibits Cx46 hemichannels through a lipid peroxide-dependent process. Hemichannels are ion channels composed of six protein subunits known as connexins (Cxs). Under physiological conditions they present a low open probability however, massive and/or prolonged hemichannel opening induces or accelerates cell death. It is known that carbon monoxide (CO) modulates many cellular processes through the activation of guanylate cyclase and/or inducing direct carbonylation of proteins by mechanisms that are either sensitive or insensitive to reducing agents. Additionally, in the article by Puebla et al. the effects of PUFA on Cx hemichannels, gap junction channels (GJCs), and channels formed by Pannexins (Panx) are reviewed. Panx as Cxs channels are permeable to large molecules such as ATP and glutamate. The authors show that PUFAs can act as either inhibitors or activators of Cxs and Panxs, and these opposing effects can be mediated by different post-translational modifications that affect these two types of proteins.

Using a different strategy, Diaz et al. have analyzed the effect of diets containing different PUFAs composition on the thermodynamic properties of Na^+^-K^+^-ATPase and on the lipid composition of gill epithelia and isolated enterocytes from euryhaline teleost fish. They demonstrate not only that branchial cells and enterocytes have different lipid composition, but also that they differently respond to severe PUFAs-deficient diets, unraveling powerful lipostatic mechanisms protecting membrane lipids in branchial cells. Paralleling these results, the thermodynamic properties of the Na^+^-K^+^-ATPase were modified in enterocytes but not in branchial cells. They discuss on direct and specific interactions of different fatty acid-containing phospholipids and cholesterol within the Na^+^-K^+^-ATPase molecular framework, determining the thermodynamic traits and enzyme activity within a given lipid microenvironment.

The original idea of this Research topic was to broaden the interest of the scientific community in studying the relationship between fatty acids (and derivatives) and integral membrane proteins. We expect this compilation may stimulate scientists to endeavor research aimed at uncovering the molecular mechanisms underlying their interaction and how it is involved in regulation/dysregulation of cell function under normal and pathological conditions.

## Author contributions

MD and MAR have edited the research topic and have written and drafted this editorial.

### Conflict of interest statement

The authors declare that the research was conducted in the absence of any commercial or financial relationships that could be construed as a potential conflict of interest.
